# Antiviral activity of *Acacia nilotica *against Hepatitis C Virus in liver infected cells

**DOI:** 10.1186/1743-422X-8-220

**Published:** 2011-05-12

**Authors:** Sidra Rehman, Usman A Ashfaq, Sana Riaz, Tariq Javed, Sheikh Riazuddin

**Affiliations:** 1 Division of Molecular Medicine, National Centre of Excellence in Molecular Biology, University of the Punjab, Lahore, Pakistan; 2 Department of Chemistry, Government College University, Lahore, Pakistan; 3 Allama Iqbal Medical College, Allama Shabir Ahmad Usmani Road, Lahore, Pakistan

## Abstract

Hepatitis C virus (HCV) belonging to the family Flaviviridae has infected 3% of the population worldwide and 6% of the population in Pakistan. The only recommended standard treatment is pegylated INF-α plus ribavirin. Due to less compatibility of the standard treatment, thirteen medicinal plants were collected from different areas of Pakistan on the basis of undocumented antiviral reports against different viral infections. Medicinal plants were air dried, extracted and screened out against HCV by infecting HCV inoculums of 3a genotype in liver cells. RT-PCR results demonstrate that acetonic and methanolic extract of *Acacia nilotica *(AN) showed more than 50% reduction at non toxic concentration. From the above results, it can be concluded that by selecting different molecular targets, specific structure-activity relationship can be achieved by doing mechanistic analysis. So, additional studies are required for the isolation and recognition of antiviral compound in AN to establish its importance as antiviral drug against HCV. For further research, we will scrutinize the synergistic effect of active antiviral compound in combination with standard PEG INF-α and ribavirin which may be helpful in exploring further gateways for antiviral therapy against HCV.

## Background

HCV is a global health problem annually affecting 180 million people worldwide [1]. Approximately 10 million (6% of the population) individuals are affected from HCV in Pakistan [2]. More than 60% of acutely infected patients turned out to be chronically infected [3]. HCV belongs to the family Flaviviridae which is enveloped viruses harboring positive-sense single-stranded RNA genome [4,5]. Whole viral genome is 9.6 kb nucleotides long encoding a large polyprotein precursor, which is cleaved by several viral and cellular proteases into different structural and non structural proteins (NS) [4,5]. Structural proteins (Core, E1, E2 and probably p7) are proteolytically slashed from the N-terminal segment of the viral polyprotein by cellular signal peptidases [6-8]. NS proteins (NS2, NS3, NS4A, NS4B, NS5A, and NS5B) are formed at the NS2-NS3 intersection by the NS2-3 metalloprotease and at the downstream position of NS3 by the NS3 serine protease [9-13].

The current standard therapy of HCV infection is pegylated interferon-α (PEG-IFN) combined with ribavirin which is recommended for 24-48 weeks depending upon viral genotype [14]. Current therapeutic options for hepatitis C are limited, especially for genotype 1. For genotypes 2 and 3, PEG INF-α plus ribavirin, can lead to a sustained virological response in up to 80% of patients [15]. In some races, response rates were still lower, with rates of approximately 25 to 30% among blacks [16]. Higher doses and prolonged therapy leading to the transient virologic response was associated with increased side-effects [17]. Hemolytic anemia is the most common side-effects of ribavirin, which has teratogenic effects [18]. Therefore, innovative, more valuable and less toxic agents are vital for HCV treatment.

HCV is becoming a serious world problem which causes acute and chronic HCV infection. Some botanical constituents such as glycyrrhizin, catechin, silymarin, phytosterols, the antioxidants *N*-acetylcysteine and vitamin E were assessed for their efficiency in curing chronic hepatitis and liver damage [19]. Therapeutic herbal extracts such as *Boswellia carterii*, *Embelia schimperi*, *Piper cubeba*, *Quercus infectoria*, *Trachyspermum ammi *and *Syzygium aromaticum *were investigated *in-vitro *for their antiviral activity against HCV protease [20]. Natural products could be enormously employed as sources for anti-HCV mediator [21]. Recently, five patients with chronic hepatitis C were treated for 1 year with *Viscum album *extract. The yield in HCV production was reduced about 6-20-fold in two patients along with stabilization of liver function, enhanced life worth and diminutive serious side effects [22]. Extract of Agaricus blazei Murill (AbM) are utilized comprehensively as a non-prescription therapy against cancer and infections, including hepatitis. The viral stack was faintly, but not drastically, lessened after 1 week of AbM treatment [23]. A diverse group of plants has been identified promisingly lowering the HCV-RNA below detection level such as cyclosporin A [24], 2-arylbenzofuran derived from *Mori cortex radicis*, as NS3 helicase inhibitor [25], Mellein, a compound extracted from the fungus *Aspergillus ochraceus*, reveals anti-HCV protease activity with an IC_50 _value of 35 μmol/L [26]. Therefore, natural plants showing antiviral activity against HCV could mark new prospects by further analysis and improvement. Various antiviral compounds possess much higher chemical assortment and biochemical precision than standard combined chemistry. Such compounds put forward foremost prospect for identifying potent structures showing antiviral activity against various targets.

In present study, we have documented AN as antiviral agent against HCV genotype 3a. Thirteen medicinal plants were collected from different areas of Pakistan. These plants were extracted in different solvents and screened out against HCV by doing real time quantification and identified AN as novel antiviral agent against HCV.

## Materials & methods

### Serum Sample collection

HCV-3a patient's serum samples used in this investigation were obtained from the CAMB (Center for Applied Molecular Biology) diagnostic laboratory, Lahore, Pakistan. Serum samples were stored at -80°C prior to viral inoculation experiments. Quantification and genotype was assessed by CAMB diagnostic laboratory, Lahore, Pakistan. Patient's written consent and approval for this study was obtained from institutional ethics committee.

### Extraction of Medicinal plants

Thirteen different plants were collected and dried on the basis of their medicinal characteristics. These indigenous plants were collected from different climatic zones of Pakistan. The plant species were correctly identified by Department of Botany, University of the Punjab, Lahore. Plants were dried under shade at room temperature. Dried parts of plants were macerated in blender, then weighed and poured methanol for overnight. Temperature should never exceed 38°C which is favorable for enzymatic activity. After 24 h solvents were filtered, residue was soaked again in methanol. Filtration was repeated over 3-4 days. Methanolic extract was further partitioned in chloroform, acetone and n-hexane. Solvents were selected on the basis of polarity for the prelude characterization of antiviral compounds. Extracts were weighed and calculating their percentage yield (data not shown).

### Stock solution preparation

50 mg of each dried plant extract was suspended in 1 ml of Dimethyl sulfoxide (DMSO) ensuing stock concentration of 50 μg/μl. Sieving the above solutions by using 0.22 um filter inside Laminar Flow Hood and stored at -20°C.

### Cell lines

The Huh-7 cell line was offered by Dr. Zafar Nawaz (Biochemistry and Molecular Biology Department, University of Miami, USA). Huh-7 cells were cultured in Dulbecco's modified Eagle medium (DMEM) supplemented with 10% fetal bovine serum & 100 IU/ml penicillin & 100 μg/ml streptomycin, at 37°C in an atmosphere of 5% CO_2_.

### MTT Assay for Toxicity

To investigate the cellular toxicity, 2 × 10^4 ^cells/well was plated into 96-well plates. After 24 h, different concentrations of Herbal extracts were added and the plate was sealed and kept at 37°C in an atmosphere of 5% CO_2 _for 24 h. After completion of extraction, media and test compounds were removed. 100 μl fresh media and 20 μl of MTT solution (5 mg/ml in PBS) were added to all wells in Columns 1-11. Wrapped the plate in aluminium foil and incubated for 3-4 h at 37°C. Media was carefully removed and added 100 μl of DMSO to dissolve the formazan crystals in Columns 1-11. MTT formazan product was determined by measuring absorbance with an enzyme-linked immunosorbent assay (ELISA) plate reader at a test wavelength of 570 nm and a reference wavelength of 620 nm.

Cell viability was obtained using the following equation.

### Anti-HCV analysis of plant extracts on Huh-7 cells

Huh-7 cell line was used to establish the in-vitro replication of HCV. A similar protocol was used for viral inoculation as established by Zekari et al. 2009 [27] and El-Awardy et al. 2006 [28]. High viral titer > 1 × 10^8 ^IU/ml from HCV patients of 3a genotype was used as principle inoculum in these experiments. Huh-7 cells were maintained in 6-well culture plates to semi-confluence, washed twice with serum-free medium, then inoculated with 500 μl (5 × 10^7^IU/well) serum media and 500 μl serum free media. Cells were maintained overnight at 37°C in 5% CO_2_. Next day, adherent cells were washed three times with 1× PBS, complete medium was added and incubation was continued for 48 hrs. Cells were harvested and assessed for viral RNA quantification by Real Time PCR. To analyze the effect of medicinal plant extracts on HCV, serum infected Huh-7 cells were again seeded after two days of infection in 24-well plates in the presence and absence of herbal extracts and grown to 80% confluence with 2 ml medium. After 24 h, cells and total RNA was isolated by using Gentra RNA isolation kit (Gentra System Pennsylvania, USA) according to the manufacturer's instructions. Briefely, cells were lysed with cell lysis solution containing 5 μl internal control (Sacace Biotechnologies Caserta, Italy). RNA pallet was solubilized in 1% DEPC (Diethyl pyrocarbonate treated water). HCV RNA quantifications were determined by Real Time PCR Smart Cycler II system (Cepheid Sunnyvale, USA) using the Sacace HCV quantitative analysis kit (Sacace Biotechnologies Caserta, Italy) according to the manufacturer's instructions.

### Formula for the calculation of HCV RNA concentration

Following formula was used to calculate the concentration HCV RNA of each sample.

IC = internal control, which is specific for each lot

## Results

Thirteen medicinal plants were collected from different areas of Pakistan on the basis of undocumented reports for antiviral screening against HCV. Plants materials were air dried and extracted in methanol. All information regarding botanical names, family vernacular names and local uses of 13 medicinal plants were shown in Table 1.

**Table 1:** List of selected Pakistani Medicinal plants.

Botanical Name	Common Name	Family	Local uses
**Withania coagulans**	Paneer dodi	Solanaceae	Remedy for dyspepsia, flatulent colic, intestinal diseases.
**Tamarix gallica**	Tamarisk, jhavuka	Tamaricaceae	Used in bleeding disorders like menorrhagia, epistaxis
**Tinospora cordifolia**	Guduchi, Tinospara, Giloy	Meninspermaceae	antipyretic, alterative, diuretic, anti-inflammatory
**Berberis aristata**	Daruhaldi, Rasvat, Maramanjal	Berberidaceae	Antimalarial, remedy for ophthalmia
**Raphanus sativus**	Radish, Daikon	Brassicaceae	As carminative, diuretic, expectorant, laxative and stomachic. Remedy for asthma and other chest complaints
**Terminalia Chebula**	Myrobalan, Hardad	Combretaceae	An effective purgative and helps in removing toxins and fats from the body
**Fumaria officinalis**	Fumitory, Earth smoke	Fumariaceae	Treatment for arthritis, liver disorders and gallstones, acts as a diuretic, slightly diaphoretic, aperient, a tonic, a digestive and as an infusion used externally in the treatment of scabies
**Kalanchoe laciniata**	Christmas tree	Crassulaceae	As topicals for ulcer, headache, emollient
**Eclipta erecta**	False daisy, Bringraj	Asteraceae	For jaundice and other ailments of the liver, spleen and gall bladder, shows antifungal activity
**Citrullus vulgaris**	Water melon	Cucurbitacea	For dropsy and hepatic congestion and intestinal catarrh
**Rheum Emodi**	Himalayan Rhubarb, Revat Chinni	Polygonaceae	As purgative and astringent tonic, strong laxative.
***Piper nigrum***	Black Pepper, Peppercorns	Piperaceae	For the treatment of pain relief, rheumatism, chills, flu, colds, increase circulation, exhaustion, muscular aches, nerve tonic and fevers
***Acacia nilotica***	Babul, Scented-pod Acacia	Fabaceae	Bark as a cough remedy, to treat eye diseases, or as a tranquillizer and even as an aphrodisiac

### Cellular Toxicity through MTT cell proliferation assay

**Figure 1:**
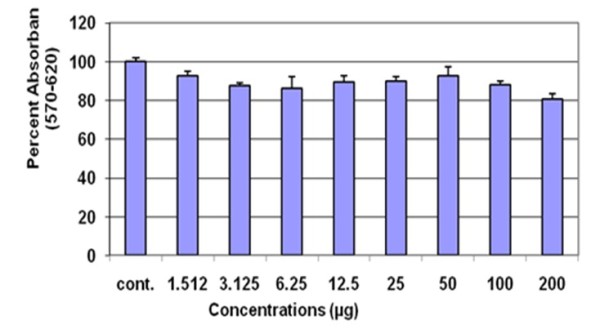
**Toxicity of extract of AN**: Huh-7 cells were incubated at different concentrations of the herbal extracts. At the end of incubation period, absorbance readings were taken through microplate sepectrophotometer

Before starting the antiviral screening against Hepatitis C virus, toxicological effects of thirteen plant extracts were determined through MTT proliferation assay. The MTT substance is reduced by mitochondrial succinic dehydrogenases in living cells to purple formazan crystals that are not soluble in aqueous water. The absorption of dissolved formazan in the visible region correlates with the number of alive cells [29]. Figure 1 exhibited cytotoxic effects of AN and demonstrated that cell proliferation of liver cells is unaffected up to a concentration of 100 μg. Similar results were observed for additional 12 medicinal extracts ranging from a concentration of 1 to 100 μg.

### Antiviral activity of methanolic extract of AN against HCV infected liver cells

**Figure 2:**
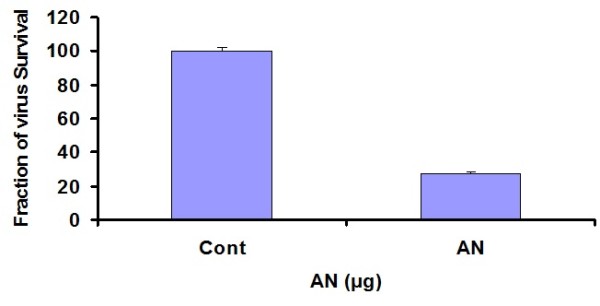
**Anti-HCV activity of methaolic extract AN**: Huh-7 cells were incubated with HCV serum and 100 μg concentration of the for 24 h. At the end of incubation period, total RNA was extracted by Gentra kit, and the levels of HCV RNA remaining were determined by the Quantitative RT-PCR assay and are shown as a percentage relative to the levels of HCV RNA in cells incubated without compound (control).

Several reports have documented that human hepatocyte cultures can be infected with HCV serum [30]. Solvent extracts from different plants were tested to determine the antiviral activity against HCV. Real time RT-PCR results showed that AN out of 13 medicinal plants exhibited antiviral effects against HCV. The results demonstrated that AN resulted in 27% inhibition of HCV RNA at non toxic concentration (Figure 2). This extract was further fractionated in different solvents on the basis of polarity.

### Characterization of methanolic extract of AN in different solvents

**Figure 3:**
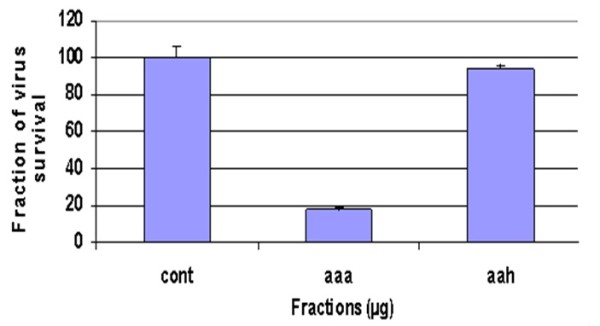
**Anti-HCV activity of acetonic and n-hexane fractions of AN**: Huh-7 cells were incubated with HCV serum and 50 μg/μl concentration of the herbal extract in different solvents for 24 hours. At the end of incubation period, total RNA was extracted by Gentra kit, and the levels of HCV RNA remaining were determined by the Quantitative RT-PCR assay and are shown as a percentage relative to the levels of HCV RNA in cells incubated without compound (control).

Further, methanolic extract of AN was fractionized by different solvents such as hexane and acetone based upon their polarity content. Results have revealed that acetone extract of an impeded viral titer to greater extent than methanolic extract at a concentration of 50 μg (Figure 3).

## Discussion

The significant prevalence of HCV infection, lack of virus-specific treatment and less compatibility of authenticated therapy suggest in exploring the medicinal plants with the aim of seeking suitable antiviral compounds. Although, agents presently in progress that target NS5B polymerase and NS3-4A protease show dominant inhibitory behavior yet all those drugs illustrate an elevated propensity for resistance sprouting. To control this dilemma, other impending viral targets need to be discovered.

In present study, we aimed at investigating antiviral compounds against HCV to identify potential bioactive agents. Thirteen plant extracts and fractions were scrutinized for their antiviral activity against HCV. A rudimentary acetonic extract of AN leaves showed evidence of exhibiting antiviral activity at a concentration of 100 μg against HCV RNA expression when evaluated via real time RT-PCR.

A lot of Acacia species have also been accounted to be useful against an array of diseases including malaria, sore throat (aerial part) and toothache (bark) [31]. Antifungal efficacy of *Acacia nilotica*, commonly called babul, have been reported recently on animals where zone of inhibition was 7.62 mm at the end of 48 h by using bark of tree containing significant amount of antimicrobial agents [32]. Significant chemopreventive and antimutagenic activity of the leaf extract of *Acacia nilotica*, on 7,12 dimethylbenz(a)anthracene (DMBA) stimulated skin papillomagenesis in male Swiss albino mice was demonstrated followed by the flower extract and then by gum [33]. AN is a source of natural antioxidant, possessing free radical scavenging activity. Ethanolic extract of AN rich in phenolic and flavonoid contents exhibiting persuasive antioxidant activity. The methanol extract of AN bark revealed the existence of Alkaloids, glycosides, tannins and phenolics which can explain its higher free radical scavenging activity and can be employed as supplement to assist the therapy of free radical induced diseases such as cancer, diabetes, inflammation, etc [34].

Prior to the appraisal of antiviral activity of selected plants, cell viability effects on Huh-7 cells were evaluated. For this rationale, cell counting and MTT calorimetric assay was employed by incubating serially diluted extracts along with Huh-7 cells. The results of cytotoxicity assessment of effective extract by using above mentioned assays found non toxic dose at a concentration of 100 μg as shown in Figure 1.

For methanolic extract from the leaves of AN, it was doable to substantiate strong antiviral activity against HCV. However, fractionation of methanolic extract of AN leaves into different solvents leads to the further specification of in-vitro antiviral activity of extract. Acetonic extract of AN leaves illustrated 82% inhibition of viral titer as compared to methanolic extract exhibiting 73% decrease in the viral RNA expression as compared to control lacking extract, as explained by the Figure 2a and 2b. Hexane fraction of AN leaves demonstrated only 7% reduction of viral titer. Results suggested the direct inactivation of HCV by using acetonic AN extract.

Natural products which are biologically dynamic in different assays are usually small molecules having both drug-like characteristics and capability of being absorbed. So the cost of formulating such medicine is much lower than that of producing from combinatorial chemistry [35]. Recently, silymarin and its two fractions (S1 and S2) are found to inhibit HCV core gene of 3a genotype and its combinational therapy with interferon will be a better option for HCV patients [36]. Globally, varieties of herbal plants have been extensively used as curative for various infectious diseases. But there is still an entailment for the identification and acquaintance of molecular targets, antiviral compound metabolism and structure-activity correlation, for engendering the medicinal plants more effectual. More importantly, it seems that auxiliary explorations are obligatory in order to depict solid conclusion.

## Abbreviations

**HCV**: Hepatitis C virus; **AN **Acacia nilotica; **PEG-INF**: Pegylated interferon; **Huh-7**: Human Hepatoma Cell line.

## Competing interests

The authors declare that they have no competing interests.

## Authors' contributions

SDR, UAA and SR contributed equally in lab work and manuscript write up. TJ helped us in chemistry techniques. All the authors read and approved the final manuscript.

## Authors' information

Sidra Rehman (MSc Chemistry), Usman Ali Ashfaq (PhD Molecular Biology), Tariq Javed (M.Phil pharmaceutical chemistry), Sana Riaz (M Phil Molecular Biology), and Sheikh Riazuddin (PhD molecular Biology and Dean Post graduate study at Allama Iqbal medical college, Lahore
